# Evaluation of a Rapid and Simple Method for Assessing Retinal Vessel Structures in Adult Zebrafish

**DOI:** 10.3390/ijms232315069

**Published:** 2022-12-01

**Authors:** Yu-Ri Lee, Myeongjoo Son, Young Sook Kim, Jin Sook Kim, Cheol-Hee Kim, Seung-Hyun Jung

**Affiliations:** 1 Korean Medicine Convergence Research Division, Korea Institute of Oriental Medicine (KIOM), Daejeon 34054, Republic of Korea; 2 Department of Biology, Chungnam National University, Daejeon 34134, Republic of Korea; 3 Research Institute for Aerospace Medicine, Inha University, Incheon 22212, Republic of Korea; 4 Julia Laboratory, Suwon 16232, Republic of Korea; 5 Department of Genetic Resources, National Marine Biodiversity Institute of Korea (MABIK), Seocheon 33662, Republic of Korea

**Keywords:** zebrafish, trypsin digestion, retinal vasculature, hyperglycemia, blood–retinal barrier, tight junction

## Abstract

The evaluation of retinal vascular structures is important for analyzing various ophthalmic diseases. Conventional trypsin digestion was used for separating retinal vasculatures in mouse, rat, and other animal models; however, the trypsin method alone is technically difficult to perform and has not been reported in zebrafish to date. In this study, we introduced a rapid and convenient method that allows the investigation of fine vessel structures at a cellular level in the relatively intact retinal vasculature of adult zebrafish. Using an anti-ZO-1 antibody, tight junction structures in retinal vessels were examined in detail and several different cell types constituting blood vessels in arterial and capillary areas were identified. In addition, using cell type-specific antibodies, we identified smooth muscle cells, blood cells, and endothelial cells in the retinal vasculature. Finally, using the hyperglycemic model, we observed the dilation of retinal vessels, the downregulation of tight junction proteins, and the reduction in smooth muscle cells. Based on these results, we provide a rapid and convenient method for the study of retinal vasculature disease in the zebrafish animal model.

## 1. Introduction

Retinal arteries and vessels are two primary blood sources in the retina. Critical blood flow to the retina occurs through blood vessels that feed the outer retina with photoreceptors. Another blood supply is provided, primarily by the retinal arteries, to feed the inner retina [1].

Retinal blood vessels are composed of various cells. Endothelial cells inside retinal blood vessels have tight junctions known to form the blood–retinal barrier. This blood–retinal barrier protects the neural retina from the leakage of toxic components from circulating blood [2]. Mural cells are vascular smooth muscle cells. These mural cells contact the endothelial cells lining the capillaries and are essential for vascular development and stability [3,4].

The identification of specific cell types and cellular structures in retinal vasculature is fundamental to understanding visual processes, retinal development, pathological progression, and therapeutic intervention [5]. Visualizing retinal vasculature is also important for the analysis of various ophthalmic diseases, including diabetic retinopathy and retinopathy of prematurity, allowing the early evaluation of vascular abnormalities, such as capillary degeneration, enlarged vessel diameter, and pericyte loss in retinal vasculature [6]. For example, blood vessels of diabetic retinopathy-suffering patients can swell and close, leading to the leakage of blood or the abnormal formation of new retinal blood vessels within the vitreous gel. These various blood vessel changes can lead to permanent vision loss and even blindness unless appropriately treated [7].

The zebrafish has gained attention as a model for studying eye diseases. Zebrafish have been extensively studied in their developmental processes and natural mutations [8]. In addition, zebrafish have been used to study human vision problems by treating them with mechanical, thermal, or chemical stimuli [9]. In addition, zebrafish have evolved as a model organism for investigating metabolic disorders, especially for hyperglycemia-induced pathologies, such as those present in diabetic patients [9,10,11,12].

To investigate retinal vasculature in disease incidence, there are several methods available that utilize elastase, lipase, collagenase, or trypsin. Representatively, the trypsin digestion technique, developed by Kuwabara and Cogan (1960), has made possible the visualization of retinal vasculature through the digestion of nonvascular components of the retina. Since then, trypsin digestion has become the standard method for analyzing retinal vasculature in rodent models. Unfortunately, the established trypsin-induced retinal digestion method is technologically challenging and can result in a high rate of specimen loss or structural infidelity [13].

Therefore, we tried to establish in this study a novel method to isolate intact retinal vasculature from adult zebrafish eyes followed by cell type-specific antibody staining and high-resolution microscopy to visualize cellular structure and morphology. We also applied this technique to analyze molecular phenotypes in a glucose-induced diabetic retinopathy model. Thus, this study could serve as a basis for further investigations attempting to characterize cellular interactions that lead to retinal vascular development.

## 2. Results

### 2.1. Trypsin Digestion and Separation of Whole Retinal Vasculature

**Figure 1:**
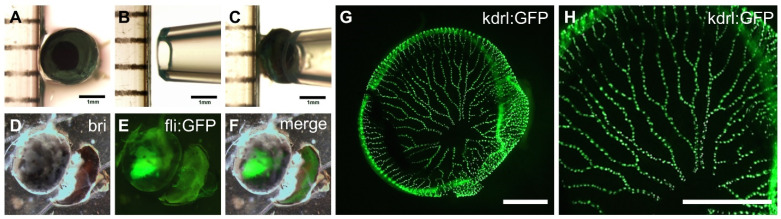
A rapid and simple method for the separation of the intact retinal vasculature from adult zebrafish eyes. (**A**–**F**) The simple procedure of detaching the whole retinal vasculature from a dissected eye using a 1 mL micropipette tip. After the removal of the lens, the intact retinal vasculature was examined by high-resolution microscopy. (**G**,**H**) Fluorescent image of whole retinal vasculature separated from adult transgenic zebrafish line *Tg[kdrl:EGFP]*, showing fine blood vessel structures. Scale bars; 1 mm for (**A**–**F**) and 200 μm for (**G**,**H**).

Although trypsin digestion is one of the most common methods to analyze retinal vasculature in experimental animal models, we newly modified a simple protocol to examine whole retinal vessels in the adult zebrafish model. First, the fixed adult zebrafish eyeball was separated, and its outer membrane was dissected. Then, the eye lens was removed to allow access to the retinal vasculature and perform enzyme digestion. After 3% trypsin treatment for 90 min, the whole retinal vasculature was carefully detached using a pipette tip (Figure 1A–F). The retinal vessel requires careful handling due to its fragility, susceptibility to damage, and ease of being misplaced given its transparency. To monitor the fine structure of retinal vessels, we used transgenic zebrafish lines, *Tg[fli:EGFP]* or *Tg[kdrl:EGFP]*, expressing green fluorescence protein (GFP) under the control of the blood vessel-specific gene promoter (Figure 1G,H).

### 2.2. Cellular Structure and Formation of Retinal Blood Vessels

**Figure 2:**
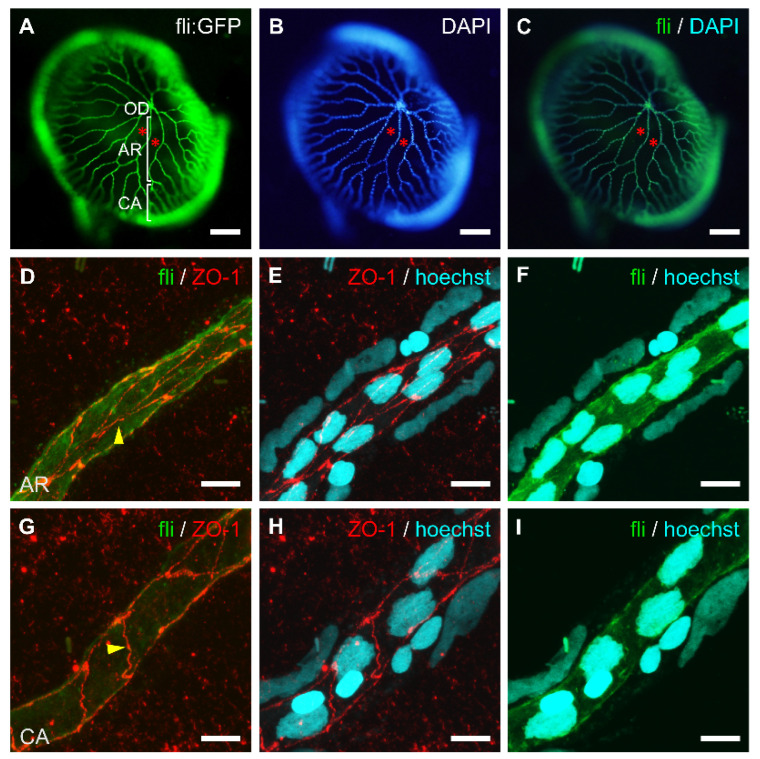
The cellular structure of whole-mount retinal vessels isolated from adult transgenic zebrafish, *Tg[fli1:EGFP]*. The vessel-specific expression of GFP (**A**) and nuclear staining with DAPI (**B**) in the retinal vasculature. (**C**) Merged image of (**A**,**B**). Retinal vessels from the optic disc (OD) branched out into the arterial region (AR) and then the capillary area (CA). Asterisks indicate membrane regions between blood vessels. (**D**–**I**) Immunofluorescent labeling of tight junction protein (ZO-1, red), nuclear staining (Hoechst, blue), and GFP expression (green) in different retinal vessel regions; arterial region (**D**–**F**) and capillary area (**G**–**I**). Endothelial cells are elongated and their long axis is parallel to the line of the blood vessel. Arrows show cellular junctions between endothelial cells. Scale bars; 150 μm for (**A**–**C**), 20 μm for (**D**–**F**), and 10 μm for (**G**–**I**).

We investigated fine vessel structures at the cellular level in the relatively intact retinal vasculature of adult zebrafish. To examine the cellular distribution in the retinal vascular tissue, we stained cellular nuclei with 4′,6-diamidino-2-phenylindole (DAPI) or Hoechst 33342. DAPI is typically used for staining the DNA content of fixed cells, whereas Hoechst is used for live cells due to its high membrane permeability. As a result, we observed that cells are mainly distributed along the line of the retinal blood vessels (Figure 2A–C).

To confirm the components of retinal endothelial cells and the expression of the tight junction protein required for blood–retinal barrier function, we examined the expression of zonula occludens (ZO-1), a tight junction-related protein, using immunofluorescence staining. As a result, ZO-1 was expressed in endothelial cells both in the arterial region and capillary area of retinal vessels. The pattern of ZO-1 expression was a zipper-like junction structure between endothelial cells in the arterial region (Figure 2D), whereas the pattern was more of a simple ladder-like structure in the capillary area (Figure 2G). The whole retinal vessel structure was examined by nuclear staining (Figure 2E,F,H,I); thus, we confirmed the differential cellular structures of the blood vessels between the arterial region and the capillary area.

### 2.3. Identification of Cell Types in Retinal Vasculature

To investigate the development of perivascular mural cells, including vascular smooth muscle cells and pericytes in the retinal vasculature, we performed antibody staining followed by confocal microscopy imaging.

**Figure 3:**
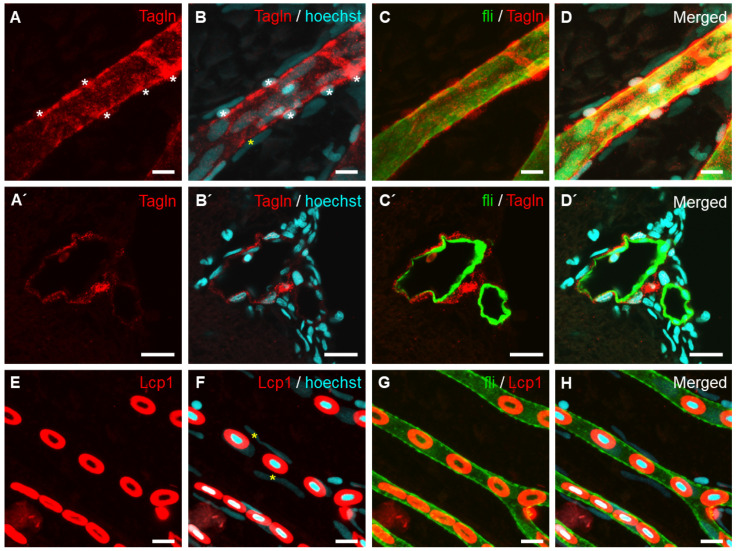
Identification of vascular smooth muscle cells in a retinal vessel of adult *Tg[fli1:EGFP]* zebrafish. (**A**–**D’**) Vascular smooth muscle cells stained with anti-Transgelin (red) are distinguishable from GFP-expressing endothelial cells (**C**,**C’**; green). (**A**–**D**) Whole-mount retinal vessel. (**A’**–**D’**) Transverse section of the eye. Cellular nuclei were stained with Hoechst 33342 (blue). (**E**–**H**) Hematopoietic cells were detected with anti-Lcp1 antibody staining (red) in the capillary region of the retinal vasculature. Asterisks indicate perivascular cells (**A**,**B**,**F**). Scale bars; 10 μm for (**A**–**D’**) and 5 μm for (**E**–**H**).

We tested with an antibody that recognizes the smooth muscle cell marker, Transgelin. Transgelin (also known as smooth muscle protein 22α, SM22α) is an abundant smooth muscle cell-specific actin-binding protein that acts as an early marker of smooth muscle cells [14]. We investigated the localization of Transgelin-positive cells in the whole-mount retinal vessels of adult zebrafish. Transgelin expression was observed to wrap vascular endothelial cells in the arterial region of the retinal vasculature (Figure 3A–D’). Interestingly, Transgelin expression was not detected in the capillary region of the retinal vasculature. To confirm vessel structure and blood flow within capillary vessels, we stained blood cells using an anti-Lymphocyte cytosolic protein 1 (Lcp1) antibody (Figure 3E–H).

**Figure 4:**
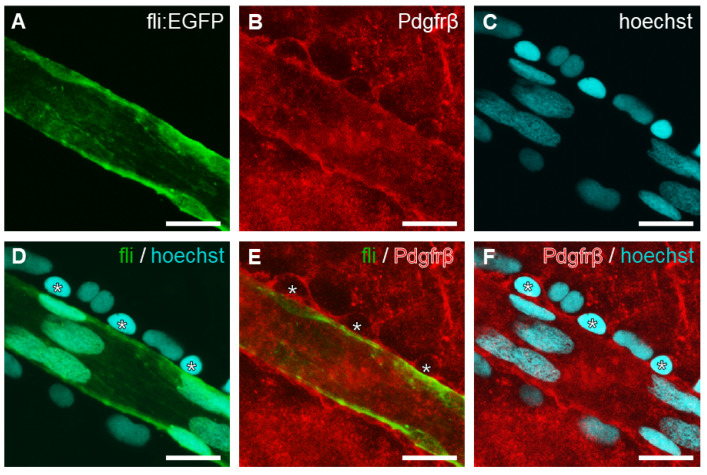
Identification of pericytes in retinal vessel structures. Confocal fluorescence image of vessel stained with anti-Pdgfrβ (**B**,**E**,**F**; red) and Hoechst 33342 (**C**,**D**,**F**; blue). Merged image of (**A**,**C**,**D**), (**A**,**B**,**E**), or (**B**,**C**,**F**), respectively. Vascular smooth muscle cells are Pdgfrβ-positive and surrounding vascular endothelial cells. Pdgfrβ-positive pericytes (asterisks) are also distinguishable from vascular endothelial cells (**D**–**F**). Scale bar, 20 μm.

To further investigate the cell type of perivascular cells, we used an antiplatelet-derived growth factor receptor beta (Pdgfrβ) antibody. Pdgfrβ-positive pericytes were found to line the outside of blood vessels (Figure 4). The presence of pericytes can differentiate the blood vessel outline from the nuclear dye signal (Figure 4D–F).

### 2.4. Changes in Retinal Vasculature in High Glucose Condition

**Figure 5:**
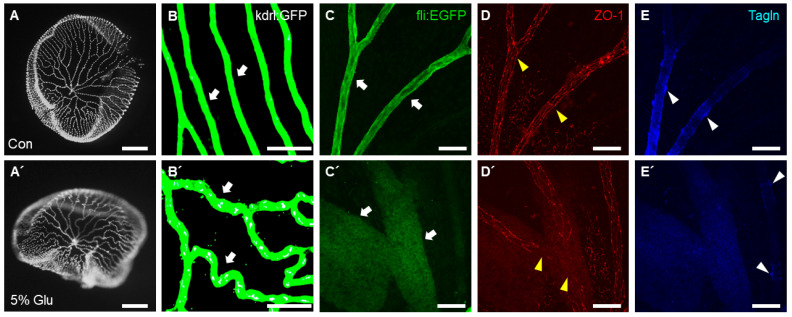
Changes in the retinal vasculature in high glucose-treated adult zebrafish. Vessel diameter was increased in high glucose-treated zebrafish (**A’**–**E’**) compared to the control (**A**–**E**). Confocal fluorescent image of vascular GFP expression (**B**,**B’**,**C**,**C’**), anti-ZO-1 (**D**,**D’**), or anti-Transgelin (**E**,**E’**) antibody staining. Arrows indicate retinal vessels. Arrowheads indicate representative cells stained with the specific antibody. Scale bars, 200 μm for (**A**,**A’**), 100 μm for (**B**,**B’**), and 50 μm for (**C**–**E**) and (**C’**–**E’**).

To study the alterations to tight junction structures and smooth muscle cell distribution in retinal vasculature under hyperglycemic conditions, we induced high glucose conditions in zebrafish by immersing the fish alternately in 5% glucose water for 30 days. The retinal vasculature of high glucose-exposed or normal fish was then isolated and immune-stained to visualize the cell structures in the retinal vessels. We observed that the vessel diameter was significantly increased in a retinal vessel from a high glucose-treated fish compared to that of control fish (Figure 5B,B’,C,C’). We found disruption of the blood–retinal barrier as confirmed by the integrity of the tight junction structure by ZO-1 expression (Figure 5D,D’). Furthermore, we investigated the effect of high glucose on retinal vessel pericyte cells by examining Transgelin expression. It was difficult to detect Transgelin-positive retinal vessel pericyte cells in the high glucose-treated zebrafish group compared to those in the control fish group (Figure 5E,E’).

## 3. Discussion

To date, there have been limited studies of retinal vessel structures at a cellular level in adult zebrafish. However, changes in structural morphology, vessel diameter, branching pattern, and tortuosity of retinal vessels have been studied as important indicators of various clinical disorders of the eye. Therefore, the visualization of intact vascular patterns and detailed cellular structure of retinal vessels is necessary for vasculopathy studies in zebrafish, including diabetic retinopathy. Although the development of hyaloid-retinal vasculature from the embryonic to the adult stage has been described by the analysis of retinal vascular structure and architecture, specific cell types and intercellular connections have not yet been identified in adult zebrafish [15,16]. This study reports a method by which the cellular structure of intact retinal vessels in adult zebrafish can be assessed and elucidated in detail. Trypsin digestion is one of the most common tools for analyzing retinal vasculature, and researchers have been utilizing this method to isolate and analyze retinal vessels in rodent animal models [17]. In this study, we established a simple method for intact retinal vasculature preparation to identify specific cell types in retinal vessels and to analyze retinal vasculopathy in adult zebrafish.

The integrity of the blood–retinal barrier has long been recognized as an important component of visual function, with its disruption leading to several retinal disorders. Macular edema results from water, albumin, and lipid leakage, causing the accumulation of lipid exudates and intraretinal fluid. The blood–retinal barrier is characterized by specific tight junctions between endothelial cells, whose function in retinal vessels is dependent on endothelial junction structure [18,19]. ZO-1 protein is located on the cytoplasmic membrane surface of intercellular tight junctions and is involved in signal transduction at cellular junctions [20]. ZO-1 plays an important role in the permeability of blood vessels in retinal vascular diseases. We identified ZO-1 expression, exhibiting a continuous zipper-like junction between endothelial cells in the retinal vasculature of adult zebrafish. Generally, endothelial cells of initial lymphatics possess discontinuous button-like junctions, whereas those of collecting lymphatics and blood vessels have continuous zipper-like junctions [21]. Both button and zipper-like junctions consist of the same adherens junction and tight junction proteins, but the proteins are arranged in distinctly different patterns [21]. In this study, we confirmed the existence of zipper-like junctions in the arterial region of retinal vessels in adult zebrafish.

The structure of retinal vessels is similar to that of vessels in other locations of the body, and vessels are of three major types: arterial, capillary, and collecting veins. Generally, blood vessels are composed of two interacting cell types: endothelial and perivascular. Endothelial cells form the inner wall of blood vessels, and perivascular cells, referred to as pericytes, vascular smooth muscle cells, or mural cells, surround the surface of blood vessels [22]. We investigated zebrafish retinal vessels at a cellular level by antibody staining and confocal microscopy imaging. In mammals, several molecular markers have been used to identify perivascular mural cells, including alpha-smooth muscle actin (SMA), Desmin, neural/glial antigen 2 (NG2), PDGFR-β, and Regulator of G-protein Signaling 5 (RGS5) [22]. However, mammalian antibodies are not useful for distinguishing different cell types as they are not conserved in zebrafish. Therefore, we selected a specific antibody that can recognize zebrafish *Transgelin* protein as a vascular smooth muscle cell marker in zebrafish. Previously, Santoro et al. reported that zebrafish *Transgelin* mRNA is expressed in the dorsal and mesenteric arteries in early-developing embryos [23,24]. Using this anti-*Transgelin* antibody, we investigated the localization of *Transgelin* protein and observed that it is expressed in vascular smooth muscle cells, wrapping blood vessels of retinal vasculature in adult zebrafish. Interestingly, *Transgelin* expression was not detected in the capillary region of retinal vessels in zebrafish, which is consistent with the results observed for mouse retinal vessels.

Wang et al. reported that a combination of a high-cholesterol diet and 3% glucose soaking could be used to induce diabetic vasculopathy in zebrafish larvae [25]. Another study proposed an animal model for nonproliferative diabetic retinopathy in adult zebrafish by immersing zebrafish in 2% glucose [10]. Our previous study also showed that the short-term exposure of zebrafish larvae to high glucose can be used for diabetic retinopathy studies [26]. To study diabetic vasculopathy in adult zebrafish, we induced hyperglycemia by alternately immersing zebrafish in a 5% glucose solution for 30 days and observed significant changes in tight junction and smooth muscle cell populations. It was observed that vessel diameter was significantly wider in retinal vessels subjected to high glucose compared to controls. We also found disruption to the blood–retinal barrier and loss of smooth muscle cells in retinal vessels after high glucose treatment. Thus, high glucose treatment can increase the permeability of retinal vessels, which could be related to diabetic retinopathy condition.

## 4. Materials and Methods

### 4.1. Zebrafish Maintenance

Zebrafish were obtained from the Zebrafish Center for Disease Modeling (Daejeon, Republic of Korea). Adult zebrafish were maintained under standard conditions at 28.5 °C with a 14 h light/10 h dark cycle [27]. Adult zebrafish were used for all experiments. All experimental protocols for animal care and use were approved by the Animal Care and Use Committee, Korea Institute of Oriental Medicine (No. 15-003), and animal husbandry and procedures were performed according to institutional guidelines.

### 4.2. Glucose Treatment

Adult transgenic zebrafish, *Tg[fli1:EGFP]* or *Tg[kdrl:EGFP]*, were placed in a 2 L cage and maintained in a 1 L water system (5 fish per 1 L) containing 5% glucose (Sigma-Aldrich, Saint Louis, MO, USA). The zebrafish were alternated every 10–14 h between a freshly prepared 5% and a 0% glucose solution. The water used in the experiments for the analysis was the same as that used in the fish facility system, except for the addition of glucose. Fish were maintained at 28 °C for 30 days.

### 4.3. Isolation of Whole-Mount Retinal Vessels

Normal or high glucose-induced adult zebrafish were fixed with 4% paraformaldehyde, stored overnight at 4 °C, and rinsed using distilled water. The fish eye was dissected and the cornea was excised along a limbal basis with fine forceps. The lens was extruded using microsurgical forceps. Dissected eyes were placed in distilled water on a 24-well plate, and water was changed every 30 min for five times. Dissected eyes were treated with 3% trypsin (0.1M Tris-HCl, pH 7.8) for 90 min at 37 °C, and rinsed three times in distilled water every hour. Retinal vasculature was carefully isolated using a 1 mL tip and pipette. Dissection experiments were performed under a stereomicroscope (SZ61, Olympus, Tokyo, Japan). Microscopy images were visualized using an Olympus stereomicroscope (SZX16, Olympus, Tokyo, Japan) or laser scanning confocal microscope (FV10i. Olympus, Tokyo, Japan).

### 4.4. Paraffin Block Preparation

Adult zebrafish were fixed in 4% paraformaldehyde for 1 day at 4 °C. After fixation, eyes were isolated and rinsed in distilled water. Paraffin sections were processed using a Paraffin-infiltration Processor (18M-T-0655; Thermo Fisher Scientific, Waltham, MA, USA) and cut to 5 μm thickness. Slides were dried, and immunofluorescence staining was performed using standard protocols [28].

### 4.5. Immunofluorescence Staining of Retinal Vessels

Whole-mount immunofluorescence staining of retinal vessels was carried out using primary antibodies against ZO-1 (1:500; Invitrogen, cat. 339100, USA), Transgelin (1:500; GeneTex, cat. GTX125994, USA), Pdgfrβ (1:500; Abcam, cat. ab107169, UK), and Lcp1 (1:500; GeneTex, cat. GTX124317, USA) [28]. For the fluorescent detection of antibody labeling, we used Alexa Fluor 568 (1:500; Thermo Fisher. Cat. No. A-11004, A-11011, USA) and Alexa Fluor 647 (1:500; Thermo Fisher. Cat. No. A32733, USA). DAPI (1:1,000; Invitrogen. Cat. No. D1306, USA) or Hoechst 33342 (1:1,000; Thermo Fisher. Cat. No. 62249) staining was used to label cell nuclei. Stained tissue was mounted on a clear glass slide and covered with a glass coverslip. Fluorescence images were examined using a confocal microscope.

## 5. Conclusions

This study provides evidence that trypsin digestion is a useful method for analyzing retinal vasculature in zebrafish, as it is in rodent models. In addition, immunofluorescence staining, flat mounts, and dye staining can allow detailed visualization of the entire retinal vascular network. We demonstrated the successful application and analysis of a new method protocol. Using a new retinal vascular separation method, retinal vessels were confirmed to have a tight junction structure using anti-ZO-1, and the presence of cells which made up the blood vessels was also confirmed with nuclear staining in the arterial and capillary areas. In addition, we identified smooth muscle cells, blood cells, and endothelial cells comprising retinal vasculature in adult zebrafish. Finally, we observed dilated retinal vessels with the disorganization of tight junctions and smooth muscle cells in the hyperglycemic zebrafish model. Based on our study, we expect investigators new to trypsin digestion to have sufficient guidance to successfully conduct and analyze retinal vasculature disease in experimental fish models, including zebrafish.

## Data Availability

Not applicable.
